# Lessons from clinical implementation of a preemptive pharmacogenetic panel as part of a testing pilot program with an employer-sponsored medical plan

**DOI:** 10.3389/fgene.2023.1249003

**Published:** 2023-08-23

**Authors:** Madeline Norris, Rachel Dalton, Benish Alam, Elizabeth Eddy, Khoa A. Nguyen, Larisa H. Cavallari, Jill Sumfest, Kristin Wiisanen, Emily J. Cicali

**Affiliations:** ^1^ Department of Pharmacotherapy and Translational Research, University of Florida College of Pharmacy, Gainesville, FL, United States; ^2^ Center for Pharmacogenomics and Precision Medicine, University of Florida, Gainesville, FL, United States; ^3^ GatorCare Health Management Corporation, University of Florida Health, Gainesville, FL, United States

**Keywords:** pharmacogenomics, implementation, pharmacogenetic panel, patient perspective, precision medicine, preemptive testing

## Abstract

**Introduction:** This manuscript reports on a pilot program focused on implementing pharmacogenetic testing within the framework of an employer-sponsored medical plan at University of Florida (UF) Health. The aim was to understand the challenges associated with program implementation and to gather insights into patient attitudes towards PGx testing.

**Methods:** The pilot program adopted a partially preemptive approach, targeting patients on current prescriptions for medications with relevant gene-drug associations. Patients were contacted via phone or through the MyChart system and offered pharmacogenetic testing with no additional direct costs.

**Results:** Of 244 eligible patients, 110 agreed to participate. However, only 61 returned the mailed DNA collection kits. Among these, 89% had at least one potentially actionable genotype-based phenotype. Post-test follow-up revealed that while the majority viewed the process positively, 71% preferred a consultation with a pharmacogenetic specialist for better understanding of their results. Barriers to implementation ranged from fatigue with the healthcare system to a lack of understanding of the pharmacogenetic testing and concerns about privacy and potential misuse of genetic data.

**Conclusion:** The findings underscore the need for clearer patient education on pharmacogenetic results and suggest the importance of the role of pharmacogenetic-trained pharmacists in delivering this education. They also highlight issues with relying on incomplete or inaccurate medication lists in patients’ electronic health record. The implementation revealed less obvious challenges, the understanding of which could be beneficial for the success of future preemptive pharmacogenetic implementation programs. The insights from the pilot program served to bridge the information gap between patients, providers, and pharmacogenetic -specialists, with the ultimate goal of improving patient care.

## Introduction

Precision medicine implementation is multifaceted, with pharmacogenetic (PGx) testing emerging as a prominent component. Over the past decade, the accessibility of PGx testing has markedly improved for patients and clinicians, driven by reduced testing costs, clinical guideline publications, and increased availability of commercial and institutional testing (Relling et al., 2020).

Despite these advancements, considerable challenges persist in delivering the benefits of PGx testing to patients. The cost of genotyping has decreased significantly over the past decade, yet a clinical PGx test may remain prohibitively expensive for many patients (Duarte et al., 2021). While insurance coverage of PGx testing has expanded, inconsistent reimbursement rates and level of coverage among payers may still serve as a relevant barrier to testing for some patients (Lemke et al., 2023). Furthermore, widespread implementation may be hampered by limited patient and provider education on PGx test utilization (Rahawi et al., 2020). Moreover, the interpretation and application of PGx test results demand expert clinical input to aid optimal clinical integration.

PGx testing is typically done reactively or preemptively. Reactive PGx testing is performed in response to initiating or planning to initiate a medication or after a patient experiences a suboptimal medication response suspected to be secondary to genetic variation (e.g., adverse event, treatment failure). Fully preemptive testing is conducted prior to the start of therapy, which may be days, months, or even years in advance of needing to use the genetic information. Preemptive testing is often done with multi-gene PGx panels, which provide lifelong results that inform prescribing decisions for a wide range of medications that may be used in the future (Greden et al., 2019). Panel-based PGx testing may also be implemented using a partially preemptive approach. With this approach, initial panel testing is reactive and performed in populations that meet specific inclusion criteria, such as patients taking a specific medication or those who may be at high risk for adverse events or treatment failure (Duarte et al., 2021). While only a subset of the genes included on a multigene panel may be necessary to address a patient’s current needs, inclusion of additional genes can inform future prescribing.

It is estimated that 9 out of 10 patients have at least one actionable phenotype relevant to current or potential future therapies (Van Driest et al., 2014; Ji et al., 2016). Despite the potential for clinical utility, many patients are not undergoing testing for various reasons, including access. Implementation of panel-based PGx testing at the payor level may improve access for health plan members and reduce costs associated with adverse drug reactions and ineffective treatments. A recent example is the Teachers’ Retirement System of the State of Kentucky, who, in partnership with Coriell Life Sciences and Know Your RX Coalition, provided PGx testing and pharmacist-led medication management services to over 5,000 of their Medicare eligible health plan members (Jarvis et al., 2022). The Teachers’ Retirement System is a state-run pension program limited to retired Kentucky public school teachers and their spouses who are Medicare eligible. Decreased costs and utilization of acute care services were both observed among their members as a result of this partnership.

In an effort to find a model that could offer an accessible option for PGx testing to their qualifying patients, University of Florida (UF) Health partnered with GatorCare, a self-funded employer-sponsored medical and pharmacy benefit plan for employees and their families at UF and UF Health Systems. Establishing a sustainable model is crucial, and implementing it through a pilot period has proven to be a successful approach (Cicali et al., 2019; Cicali et al., 2023). We implemented a pilot where panel-based PGx testing was provided without direct costs to patients and buccal swab DNA collection kits were mailed to the participant’s home. The overall purpose of this pilot was to develop a feasible method for providing partially preemptive PGx testing to UF Health patients during the coronavirus (COVID-19) pandemic. In order to improve PGx panel testing implementation models, both successes and challenges encountered are described. Additional implementation metrics were collected to gain an understanding of the real and perceived clinical usefulness of partial preemptive PGx panel testing.

## Methods

### Genotyping

PGx testing was performed with GatorPGx, a panel-based PGx test offered by UF Health’s internal laboratory, which is a College of American Pathologists/Clinical Laboratory Improvement Amendments (CAP/CLIA) certified laboratory. The GatorPGx panel tests for 8 pharmacogenes (*CYP2C19, CYP2D6, CYP2C9, CYP3A5, SLCO1B1, CYP2C* cluster*, CYP4F2,* and *VKORC1*) using the QuantStudio 12K Flex Real Time PCR System (Applied Biosystems by Life Technologies) and Life technology TaqMan^®^ SNP Genotyping Assays (Marrero et al., 2020). The GatorPGx panel has the capability to detect CYP2D6 copy number variations, however, it is not able to specify which allele nor how many copies are present. A full list of the individual star alleles and single nucleotide polymorphisms tested for can be found in Supplementary Figure S3. Phenotypes were derived based on guidance from The Clinical Pharmacogenetics Implementation Consortium (CPIC). Of note, previous activity score cutoffs for defining CYP2D6 metabolizer phenotypes are reported as these were used at the time of this implementation (i.e., activity score of 1.0 is defined as normal metabolizer). (Caudle et al., 2020).

### Patient eligibility and enrollment

Published Clinical Pharmacogenetics Implementation Consortium (CPIC) guidelines and previously implemented gene-drug pairs at UF Health were used to curate a list of 27 medications that have clinically relevant pharmacogenetic association(s) with a gene included in the GatorPGx panel. For this paper, these 27 medications are referred to as “panel drugs; ” a complete list of panel drugs can be found in Table 1.

**TABLE 1:** Panel drugs (*n* = 27).

Pain	Relevant Gene(s)	Mental health	Relevant Gene(s)
Celecoxib, ibuprofen, flurbiprofen, meloxicam, piroxicam	CYP2C9	Atomoxetine, fluvoxamine, paroxetine	CYP2D6
Codeine, hydrocodone, tramadol	CYP2D6	Citalopram, escitalopram, sertraline	CYP2C19

GERD/H.Pylori	Relevant Gene	Other	Relevant Gene(s)
Dexlansoprazole, esomeprazole, lansoprazole, omeprazole, pantoprazole, rabeprazole	CYP2C19	Tacrolimus	CYP3A5
		Tamoxifen	CYP2D6

Cardiovascular	Relevant Gene(s)		
Clopidogrel	CYP2C19	Phenytoin	CYP2C9
Simvastatin	SLCO1B1	Voriconazole	CYP2C19
Warfarin	CYP2C9, VKORC1 CYP2C cluster		

**FIGURE 1:**
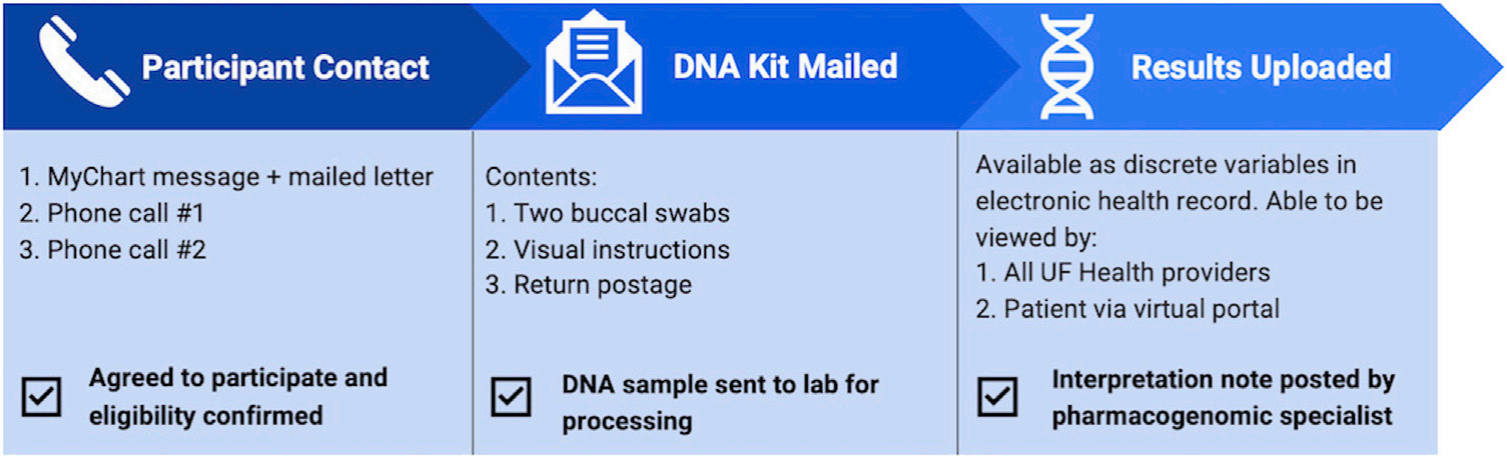
Flow of patient experience throughout the pilot program. Eligibility was determined based on review of electronic health record, then confirmed with the patient once contact was established. Upon accepting participation in the pilot, patients were mailed a DNA collection kit and once returned, pharmacogenetic results were uploaded to the electronic health record.

UF Health patients were able to participate in the pilot if they were: 18 years old and older, had active enrollment in a GatorCare benefit plan, an active prescription for a panel drug regardless of duration of therapy, and at least one outpatient visit at UF Health in the preceding 12 months. The pilot was registered with UF Health as a quality improvement project. Authors reviewed the electronic health record (EHR) to identify eligible patients and contacted relevant providers at various UF Health clinics to opt out if they did not want their patients to participate or be contacted by the pilot program. Participating UF Health providers included 19 family medicine providers, 13 gastroenterologists, and 3 internal medicine providers. Providers were also given the opportunity to self-identify patients who may benefit from testing and offer enrollment in person, so long as they fit eligibility criteria. If they desired to do so, instructions and materials for DNA sample collection were given to the respective provider. Patients were offered the opportunity to participate between October 2020 and March 2021. Eligible patients were contacted both through MyChart messages and mailed letters (Figure 1). The outreach information included educational PGx information and described the process of undergoing PGx testing through the pilot program (Supplementary Figures S1, S2). If there was no response to the message or mailed letter, two follow-up phone calls were performed, each at least 1 week apart. Once contact was established, medications and benefit plan enrollment were confirmed directly with the patient to verify eligibility for the pilot. If the patient declined participation, the reason was documented. If the patient did not respond after two letters and two follow-up phone calls, they were assumed to have declined participation.

### Sample collection and results

If the patient agreed to participate in the pilot, a buccal DNA collection kit was mailed directly to the patient, which included a pre-paid mailing envelope to return the sample to the laboratory. If patient was identified and agreed to participate in person, the buccal sample was collected in clinic. After processing the samples and running the GatorPGx assay, the laboratory returned the genotypes and phenotypes to the EHR as discrete variables. Once available in the EHR, the pharmacy team was notified, UF Health providers were able to review the results in Epic, Best Practice Alerts (BPAs) were able to fire, and patients were able to review results via MyChart. Upon pharmacy notification that the results were returned, a pharmacogenetics-trained pharmacist reviewed the results within 24–48 h and wrote a consult note that included interpretations of the patient-specific genotype/phenotype and recommendations for current and future medications. The consult note was documented in the EHR and routed to the participant’s provider via Epic in-basket message.

### Post-test data analysis

Patients who completed PGx testing received a post-test follow-up phone call to inquire about overall satisfaction and perception of the pilot program, as well as to obtain perspectives on the clinical utility of testing. Post-test follow-up phone calls were attempted between September 2021 through March 2022 and occurred a minimum of 3 months after patients’ PGx test results were resulted to the EHR. If contact was successful, patients were asked the following questions and asked for a verbal response: 1) *What is your overall opinion of the process? 2*) *Would you have preferred a follow-up appointment with a PGx specialist to explain the results? 3*) *Do you feel like these PGx results may have a potential impact on your care?* Any additional comments made by the patient relating to their experience or perspective on the program were documented. Data were analyzed descriptively (mean ± standard deviation or as frequencies), with pairwise comparisons performed using Student’s t-test. Metrics collected to gain an understanding of the real and perceived clinical usefulness of partial preemptive PGx panel testing included participant demographics, potential genotype actionability, current actionability of gene-drug pairs, prevalence of drug-drug-gene interactions that affect CYP2D6 clinical phenotype (phenoconversion), and post-test participant perspectives. When assessing potential actionability, genotypes were considered actionable if a potential drug or dose change was suggested by CPIC guideline recommendations for any medication, regardless of what medications the participant was currently taking. Current actionability of gene-drug pairs were determined based on the participant’s clinical phenotype and currently active panel drug; if the pair’s associated guideline recommendations published by CPIC suggested a drug or dose change, it was considered actionable.

## Results

**FIGURE 2:**
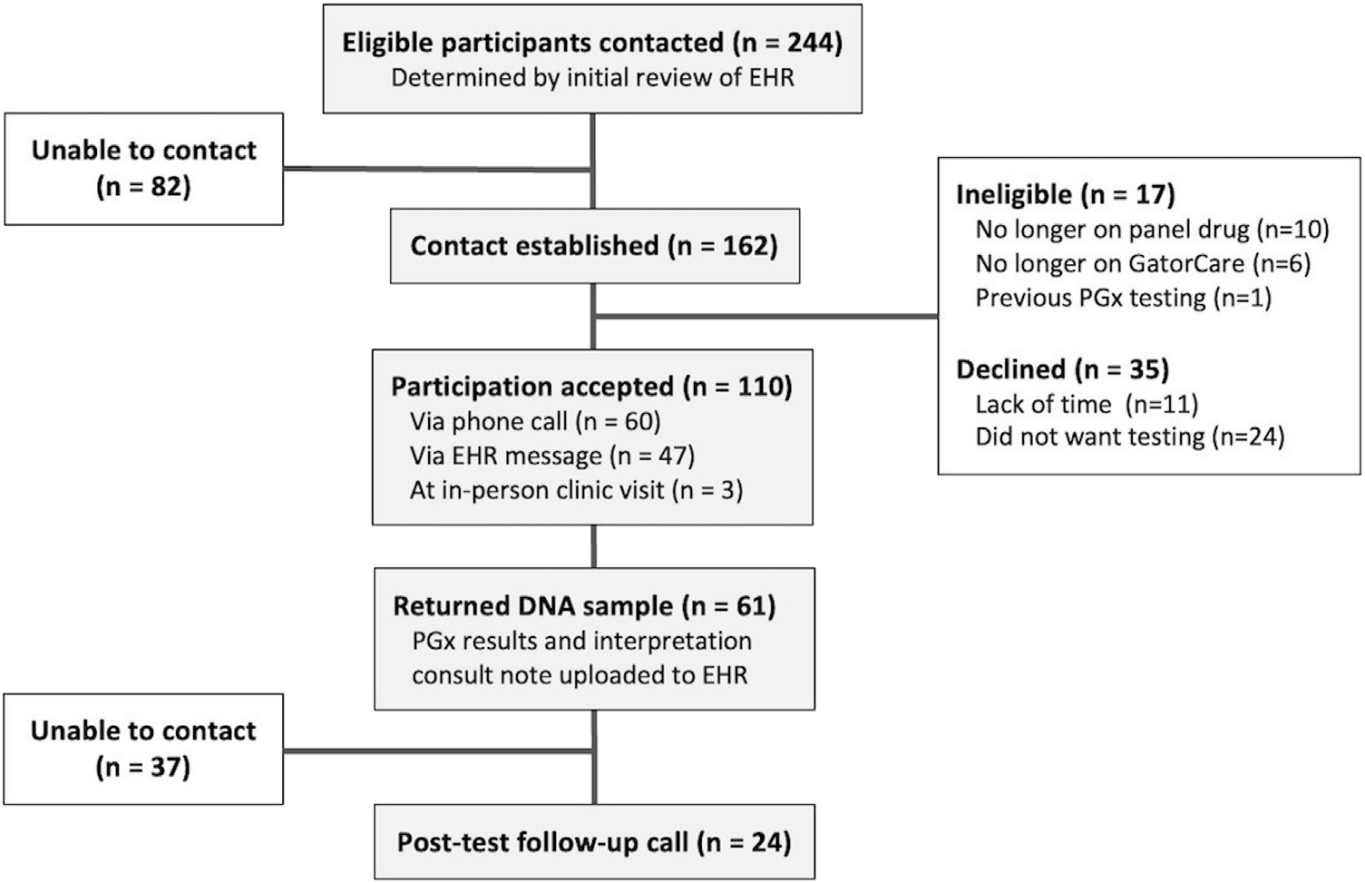
Patient participation and involvement. Eligibility criteria included actively prescribed a panel drug, currently a GatorCare benefit plan member, and having had at least one clinic visit within UF Health in the last year.

A total of 244 eligible UF Health patients were identified through initial EHR review. Contact was successfully established with 66% (*n* = 162) of these patients (Figure 2). Upon establishing contact and confirming eligibility, 17 patients were excluded from the pilot as they were no longer on a panel drug (*n* = 10), no longer enrolled in a GatorCare benefit plan (*n* = 6), or already had PGx testing (*n* = 1). Of the 145 confirmed eligible patients, 76% (*n* = 110) agreed to participate. The majority of patients (55%; *n* = 60) agreed to participate through a phone call; 50 agreed to participate after the initial phone call, and an additional 10 patients agreed after a follow-up call. Most other patients (43%; *n* = 47) agreed to participate in the pilot by responding to the initial MyChart message. One provider requested DNA sample collection materials at their clinic as they opted to self-identify eligible patients. The remaining 3% (*n* = 3) agreed to participation while at an in-person UF Health clinic visit with this provider after eligibility was confirmed. No patients opted to participate as a direct result of the physically mailed letter. The remaining 24% (*n* = 35) of the confirmed eligible patients with whom we established contact declined participation. Many of those who declined expressed a fatigue with the healthcare system explaining that they simply did not have time for more testing, while others simply did not want to participate in PGx testing, especially if they did not understand it.

Of the 110 patients who opted to participate, 55% (*n* = 61) provided their PGx sample and received PGx results. The majority of these patients (*n* = 58) returned their mailed DNA collection kit, while those enrolled in person completed testing in person the same day. The average turnaround time from outbound mailing of DNA collection kits to the date the results were reported was just over 1 month (30.9 ± 14.6 days), and some (*n* = 3) outliers took up to 3–4 months to return their DNA collection kits. Once returned and delivered to the UF Health Pathology lab, the average turnaround time for sequencing is 3–7 days. Patients who provided their DNA sample were mostly female (64%), White (87%), and were an average of 51 years old (Table 2). All 61 patients received PGx results for the 8 genes included on the panel except for one patient whose CYP2D6 genotype returned as “indeterminate.” Nearly 89% of patients had a minimum of one potentially actionable phenotype as determined by their genotype-based phenotype.

**TABLE 2:** Patient demographics.

Demographic	*n* (%) total *n* = 61
Age (years)	
Minimum	25
Mean (SD)	50.93 ± 10.21
Maximum	71
Sex	
Female	39 (63.9%)
Male	22 (36.1%)
Race	
White	53 (86.9%)
Asian	3 (4.9%)
Other	3 (4.9%)
Black or African American	2 (3.3%)
Ethnicity	
Not Hispanic or Latino	59 (96.7%)
Hispanic or Latino	1 (1.6%)
Unknown/Not Reported	1 (1.6%)

**FIGURE 3:**
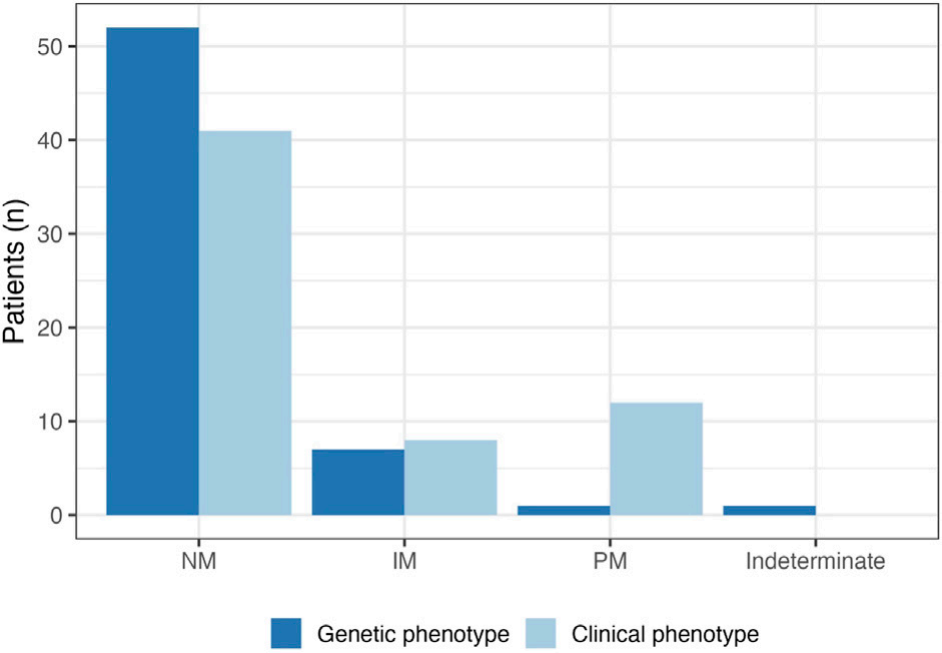
CYP2D6 genotype-based and clinical phenotype. A total of 14 patients were taking strong or moderate CYP2D6 inhibitors. Almost all (*n* = 13) experienced phenoconversion to intermediate metabolizer (IM) or poor metabolizer (PM) as a result; clinical phenotypes are represented by the light blue bars. The prevalence of CYP2D6 PMs increased from 1.5% to 20% after accounting for phenoconversion.

A complete breakdown of participant genotype/phenotype results can be found in Table 3. A total of 23% of patients (*n* = 14) were taking a strong or moderate CYP2D6 inhibitor, and all but one participant experienced phenoconversion of their genotype-based phenotype to a clinical phenotype of either CYP2D6 poor or intermediate metabolizers as a result (Figure 3).

**TABLE 3:** Pharmacogenetic results.

Phenotype/Genotype	*n* (%) total *n* = 61
CYP2C9	
IM	25 (41%)
NM	36 (59%)
CYP2C19	
PM	2 (3%)
IM	12 (20%)
NM	30 (49%)
RM	16 (26%)
UM	1 (1.5%)
CYP2C Cluster	
G/G	46 (77%)
G/A	13 (21%)
A/A	2 (2%)
CYP2D6	
PM	1 (1.5%)
IM	7 (11%)
NM	52 (85%)
Unable to genotype	1 (1.5%)
CYP3A5	
NM	11 (18%)
PM	50 (82%)
CYP4F2	
*1/*1	27 (44%)
*1/*3	27 (44%)
*3/*3	7 (11%)
SLCO1B1	
Decreased function	15 (24.5%)
Normal function	46 (75.5%)
VKORC1	
G/G	22 (36%)
G/A	27 (44%)
A/A	12 (20%)

PM, poor metabolizer; IM, intermediate metabolizer; NM, normal metabolizer; RM, rapid metabolizer; UM, ultrarapid metabolizer

**FIGURE 4:**
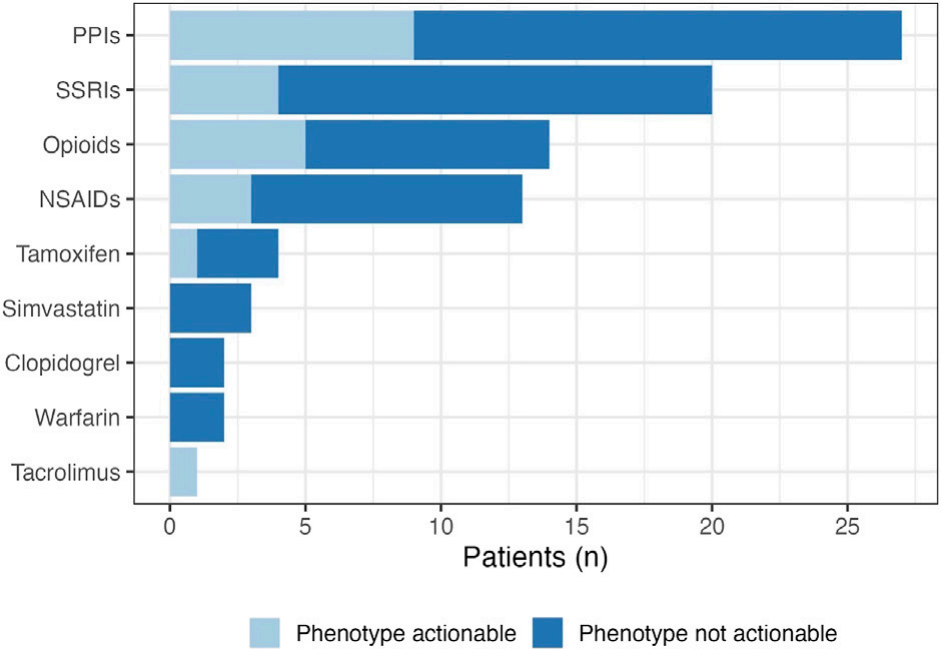
Pharmacogenetic drug class at baseline. The prevalence of actionable gene-drug pairs are represented by the light blue section of the bars. Actionability was determined based on the patient’s clinical phenotype, and the associated CPIC guideline recommendations for relevant panel drugs.

Of the 61 patients who participated, there was a combined total of 86 active panel drug prescriptions at time of enrollment. Most patients were on one panel drug, however nearly 40% of patients had at least two active panel drugs in their medication list at baseline. It is worth noting that three patients thought to be eligible were realized to be on zero active panel drugs following the return of their DNA collection kit. This was largely due to patients being on topical tacrolimus rather than the oral formulation which was reported by these patients during telephone follow-up conversations. The most common drug class of active panel drugs were proton pump inhibitors (PPIs; *n* = 27 patients with an active PPI prescription), followed by selective serotonin reuptake inhibitors (*n* = 20). Pain management was also relevant with 14 active panel drugs being opioids, and 13 being nonsteroidal anti-inflammatory drugs (Figure 4). The most common active panel drug was omeprazole (*n* = 14), followed by tramadol (*n* = 9), escitalopram (*n* = 8) and pantoprazole (*n* = 8). Just over a quarter (*n* = 23) of gene-drug pairs were currently actionable, seven of which have at least a moderate level of evidence supporting them. The most common actionable gene-drug pair (*n* = 5) included omeprazole in CYP2C19 poor or intermediate metabolizers. This is followed closely (*n* = 4) by pantoprazole in CYP2C19 intermediate or ultrarapid metabolizers. An additional 16% of active panel drugs (*n* = 14) were PPIs in patients who are CYP2C19 normal or rapid metabolizers. This gene-drug pair is worth noting as it may become actionable in the future based on indication (Lima et al., 2021).

**FIGURE 5:**
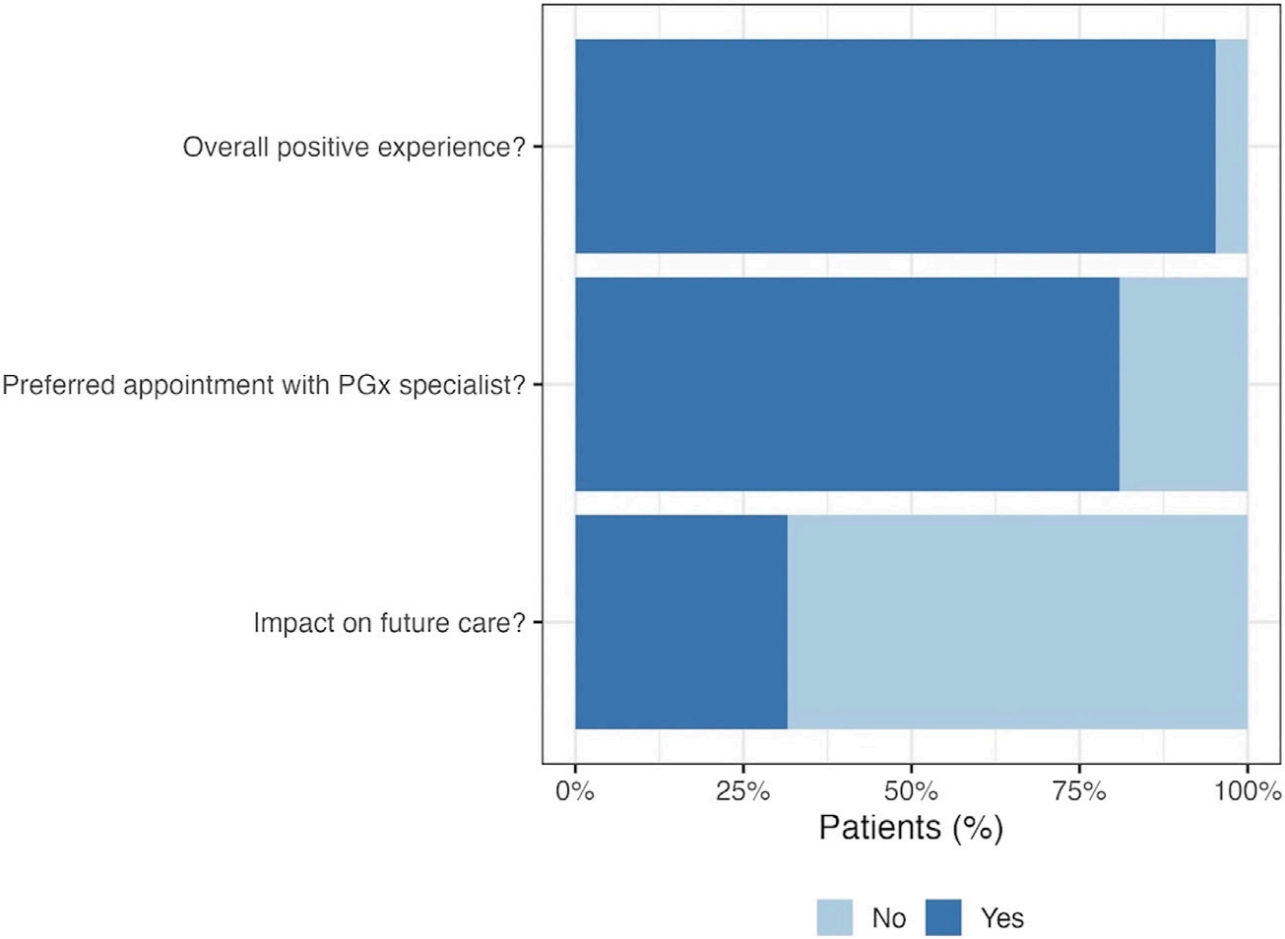
Patient perspective at post-test call. Post-test follow up phone calls were made to patients with pharmacogenetic results in order to gain insight into participant perceptions on the pilot program. Twenty-four patients completed the post-test call, the majority of which expressed preferring to have had an appointment with a PGx specialist following testing.

Post-test follow-up phone calls were successful with 39% (*n* = 24) of patients with PGx results. As displayed in Figure 5, follow-up calls revealed that more than 80% of patients would describe the process as an overall positive experience, but 71% said they would have preferred an appointment with a PGx specialist to help explain and interpret their genetic results. Only 25% of contacted patients expressed feeling like their PGx test results may have a potential impact on their future care, and many of those who did not perceive a potential impact noted they and/or their physician did not understand their results.

## Discussion

This implementation pilot provided new insights into patient attitudes about PGx testing and the specific challenges of implementing a PGx testing program through an employer-sponsored medical plan. Among the eligible UF Health patients screened for inclusion in the pilot, 76% were offered PGx testing with no additional direct costs. Those who declined to participate cited reasons such as lack of time or interest, uncertainty about the benefits of PGx testing, and concerns about privacy and the potential for misuse of their genetic data. This suggests that, from a patient’s perspective, cost is not the only relevant barrier to implementing PGx testing. Panel-based PGx testing was ordered using a partially preemptive approach, in which we targeted patients with current prescription(s) for medications with relevant gene-drug associations. While this approach shares elements with reactive testing, it differs in that patients tested reactively are often at risk for or have experienced an adverse event or treatment failure, while patients tested using a partial preemptive approach may not have been having any issues with their medications. This may have diminished the urgency of completing the test. These results suggest that preemptive testing could potentially serve as a barrier to PGx testing based on lack of understanding of the test’s purpose by patients and providers. Although we aimed to take cost, uncertainty about insurance coverage, and in-person clinic visits out of the equation, less obvious implementation barriers were revealed in this implementation. These challenges, however, help identify important pitfalls that can be useful for the success of future preemptive PGx implementation programs. A description of implementation challenges encountered and lessons learned are described in Table 4.

**TABLE 4:** Summary of relevant implementation challenges encountered and potential solutions.

Category	Challenge	Potential solution or lesson
Patient participation	Limited understanding of the purpose of testing • Low return rate of DNA collection kits	• Enhanced pre-test education for patients is needed. This would provide more background information explaining who can benefit and which medications are informed by testing
		• Tests were provided free of charge. Patients may respond better with a financial or other type of buy-in to the testing process
		• Post-test patient-friendly educational handouts describing results help bridge this knowledge gap with patients
	Low participation rate • Response to participation requests was relatively low • Post-test calls were only successfully completed in a handful of patients	• A verbal conversation can help mitigate patients’ concerns with testing. The majority of patients with established contact did ultimately agree to participate
		• Pre-emptive testing may inherently serve as a barrier to implementation. Without any current drug therapy problems, the purpose of testing may be unclear
		• Collecting information on disease state and current response to medications in future implementation projects would provide insight into which patients are more likely to participate in PGx testing
	Lack of perceived benefit • Low perceived impact on care	• Improving post-test education by offering consultation with a pharmacist, for example, could improve perceived benefit of testing
		• While most participants did not think PGx testing would have an impact on their care, it is encouraging to see the majority had a desire to understand their results better
Provider education	Knowledge gaps and varying levels of comfort with interpreting results • Some patients reported their provider did not know how to use their PGx results	• Utilizing pharmacist consult notes to help providers use PGx information with more confidence is needed
		• Having a mechanism for requesting a consult or pharmacogenetic test interpretation integrated into the electronic health system is also beneficial
EHR	EHR-reported medication lists are often inaccurate or incomplete	• Patient engagement is needed to obtain an accurate medication history in most EHR systems. This can improve the ability to provide relevant medication recommendations
	The system may not be set up to access pharmacogenetic results easily	• Pharmacogenetic results were previously only reported as a lab value, which is not optimal if a non-expert is trying to interpret results. Our institution has since implemented a separate tab within the EHR entitled “Genomic Indicators”. Providers can now use this tab to view patient-specific recommendations for medications informed by PGx testing

*EHR,* electronic health record; *BPA*, best practice advisory.

While the patients may not have had any known medication-related problems at the time of test initiation, the purpose of this pilot was to complete testing so that the results would be available should the patient need them in the future. It is highly likely that the results will be impactful in the future as 89% of patients had one potentially actionable genotype-based phenotype. While the majority of tested patients have a clinically useful PGx test result, only 25% of patients contacted for follow-up expressed feeling that their results would have an impact on their future care. This suggests that patients will miss out on the benefits of PGx testing if they do not understand what their results mean. Patients noted that they did see their results in their patient portal, but many expressed not understanding the results. At the time of this implementation patient-friendly language around the results in the patient portal did not exist; we have since updated the portal to provide basic information. Even with this update, it is most beneficial for a patient to be educated on their results by a PGx-specialist, or their prescriber, if knowledgeable about PGx results. Some patients revealed that they did discuss the PGx results with their primary care physician, but their physician did not understand the results enough to apply them to their care. Although a consult note was placed in patients’ EHRs based on their current medication lists and genetic results, most patients still expressed that they would have preferred an opportunity to speak with a PGx-trained pharmacist to help understand results. Additionally, more than 20% of patients with PGx test results were expected to experience drug-induced CYP2D6 phenoconversion based on their medication lists. The prevalence of phenoconversion further emphasizes the need for PGx-trained pharmacists to help with the accurate interpretation of genetic results that takes concomitant therapy into account.

Another challenge encountered was the reliance on incomplete or inaccurate medication lists in patients’ EHRs. Despite confirming eligibility at time of initial contact, the process failed to identify all ineligible patients. Three patients thought to be eligible were enrolled and received pharmacogenetic testing, but it was later realized that these patients did not take any panel drugs. This suggests that automating phenoconversion would not be feasible and further supports pharmacist interpretations. The use of EHR medication lists also has the potential to miss any over-the-counter medications affected by pharmacogenetics, such as certain NSAIDs or PPIs. These issues highlight the importance of engaging with patients to provide them with accurate and relevant interpretation of PGx test results. Engaging with patients earlier could potentially confirm medication lists, but this is not always possible in an automated workflow.

There was a clear desire by patients to better understand their genetic results and relevant medications is not novel and is indicative of a need for the expansion of pharmacist-led PGx clinics like those already being implemented at several institutions across the United States (Duarte et al., 2021). In fact, since the completion of this pilot program the UF College of Pharmacy and Center for Pharmacogenomics and Precision Medicine have partnered with UF Health to launch MyRx, 2022 (https://myrx.ufhealth.org/), a clinical PGx consultation service utilizing online visits with PGx-trained pharmacists to educate patients on their test results.

Implementations such as this pilot program serve as an important tool for identifying ways to bridge the information gap between patients, providers, and PGx-specialists. This engagement may continue to become easier as the evidence base supporting preemptive or partially preemptive PGx testing’s impact on clinical outcomes continues to grow. The recently published PREPARE study conducted a large, open-label, prospective, cluster-randomized-controlled implementation study of a 12-gene PGx panel (Swen et al., 2023). The investigators observed a 30% reduction in patient-reported clinically relevant adverse drug reactions with the use of PGx-guided prescribing. The PREPARE study included close to 7,000 patients; however, study results are limited by a lack of diversity. There is currently still a need for more diverse PGx implementation studies to properly identify and address implementation barriers unique to these populations.

### Limitations

Lack of diversity is a common limitation in pharmacogenetic trials, and is relevant to our pilot program, as well. With an already relatively small sample size and 87% of enrolled patients being White, it is likely that the experiences of underrepresented populations in the United States are not well represented by our patient population. The small sample size of this pilot program could have limited our ability to fully assess the challenges identified above. A larger patient population in future implementation programs would likely provide further insight into relevant challenges. Additionally, because this pilot was conducted during the COVID-19 pandemic, patient attitudes and behaviors may have limited generalizability due to potential hardships associated with the pandemic.

There are several limitations to the GatorPGx Panel used for pharmacogenetic testing in this pilot. Eight genes were included in the panel that were thought to have a potential impact on a large majority of patients. However, there are genes with a high level of evidence and prescribing guidance available that were not tested for (e.g., DPYD, UGT1A1). It is also possible that some patients may have had a single nucleotide polymorphism (SNP) present that went undetected by the GatorPGx Panel. Only SNP’s on a predetermined list associated with the assay (Supplementary Figure S3) were able to be detected. If one of the listed SNPs was not identified, then the resulting genotype would default to the wildtype “*1” allele. This has potential to misclassify patients with altered enzyme function as a normal metabolizer.

The curated list of panel drugs resulted in relevant limitations to this pilot program, as well. Not all relevant medications (e.g., atorvastatin) that can be informed by pharmacogenetic testing were included, and therefore some patients who were on a medication relevant to PGx may have been missed. Additionally, certain panel drugs (e.g., rabeprazole) are no longer considered to be informed by pharmacogenetic testing based on updated evidence and CPIC guidelines published after the start of this pilot program (Lima et al., 2021; Cicali et al., 2023).

There are several limitations regarding the enrollment, contact, and follow-up in this pilot. Eligibility was determined based on active medications in the patient’s EHR, which may have been inaccurate or incomplete. Inaccurate medication lists may have resulted in the exclusion of patients who were actually eligible, or *vice versa*. All follow-up phone calls were conducted and evaluated by a single author, which may limit the validity of their interpretation. Although no formal parameters were set up to guide the evaluation of patient responses, the same three questions were asked to everyone and an effort was made to keep consistency. Regardless of these described limitations, we were still able to learn key lessons from this pilot implementation to guide future implementations.

## Data Availability

The raw data supporting the conclusion of this article will be made available by the authors, without undue reservation.
