# Supplementary material for: Lessons from clinical implementation of a preemptive pharmacogenetic panel as part of a testing pilot program with an employer-sponsored medical plan
Source: Front Genet. 2023 Aug 23;14:1249003. doi: 10.3389/fgene.2023.1249003 (PMC10482099; doi:10.3389/fgene.2023.1249003)
Supplement: Supplementary file 1 [file Image1.pdf]

Supplementary Material

Figure S1: Examples of Educational Information Provided to UF Health Patients When Offering Participation in the Pilot Program

What is pharmacogenetic testing?
Your genes (DNA) can affect your response to some medicines. Pharmacogenetic tests identify differences in genes that may affect how a medicine works for you. Many factors affect which medicines your provider may choose for you such as your allergies, other medicines, and other health problems. This testing may help you and your provider decide which medicines and doses work best for you. Knowing this information may help reduce side effects, increase your chances of successful treatment, and help you achieve better health outcomes.

What genes will be tested and how will your DNA be collected?
If you decide that testing is right for you, you will have the opportunity to receive the UF GatorPGx panel, which tests for genes that affect some medications for treating depression, anxiety, heartburn, pain, and certain medications used after a heart attack. Your DNA will be collected using a cheek (buccal) swab, which is an easy and relatively non-invasive way to collect DNA from the cells on the inside of your cheek.

If you are interested in being tested, what are the next steps?
If you are interested in receiving pharmacogenetic testing, please reply to your MyChart message or call us at the number below to indicate your interest. Someone will contact you with instructions and you will receive a DNA collection kit with a prepaid envelope in the mail. If you would prefer to come in for a clinic visit, we will call you to arrange a date and time for you to come to a UF Health facility to provide your DNA sample and participate in a telehealth visit if you wish. Once we receive your DNA sample, the UF Health Pathology Laboratories will process your genetic results and securely result them into your electronic medical record for your medical team.

How do you access your results?
You will be able to access your results through MyChart. If after reviewing your result you have questions, you can call 352-273-6415 and you will be assisted with any questions you may have.

How is your health information be protected?
We follow required HIPAA (Health Insurance Portability and Accountability Act) and GINA (Genetic Information Nondiscrimination Act) guidelines and practices.

Figure S2: Process for Undergoing Pharmacogenetic Testing as a Part of the Pilot Program

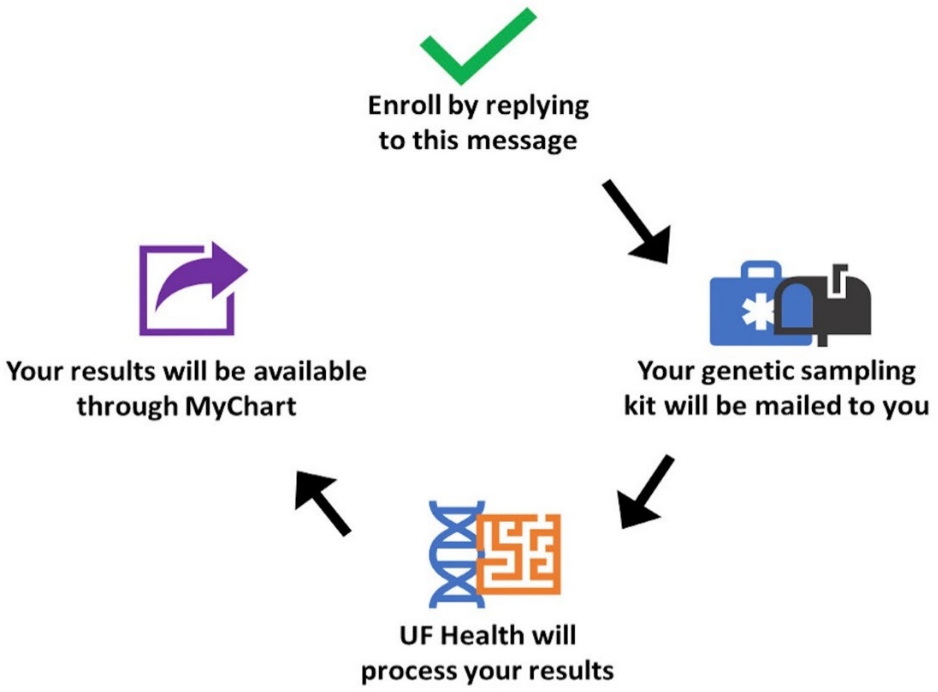

Figure S3. GatorPGx Pharmacogenetic Panel

Gene Symbol	SNP rs#	Star Allele
**CYP2C19**	rs4244285	*2
	rs4986893	*3
	rs28399504	*4
	rs72552267	*6
	rs41291556	*8
	rs6413438	*10
	rs12248560	*17
**CYP2C9**	rs1799853	*2
	rs1057910	*3
	rs28371686	*5
	rs9332131	*6
	rs7900194	*8
	rs28371685	*11
**CYP2C Cluster**	rs12777823	N/A
**CYP2D6***	rs16947	*2
	rs35742686	*3
	rs3892097	*4
	rs5030655	*6
	rs5030867	*7
	rs5030865	*8
	rs5030656	*9
	rs1065852	*10
	rs28371706	*17
	rs59421388	*29
	rs28371725	*41
**CYP3A5**	rs776746	*3
	rs10264272	*6
	rs41303343	*7
**CYP4F2**	rs2108622	N/A
**SLCO1B1**	rs4149056	*5
**VKORC1**	rs9923231	N/A
*CYP2D6 gene rearrangements associated with gene deletion (*5) and duplication (DUP) genotypes, which are defined as two or more gene copies per allele (copy number) were also tested for.		

SNP, single-nucleotide polymorphism; N/A, not applicable
